# Supplementary material for: Are carbon emissions trading and green financial instruments synergistic? -Comprehensive quantitative research based on content analysis
Source: PLoS One. 2024 Mar 7;19(3):e0298601. doi: 10.1371/journal.pone.0298601 (PMC10919601; doi:10.1371/journal.pone.0298601)
Supplement: S1 Data — (ZIP) [file pone.0298601.s001.zip › Data/地级市Z_F工作报告.zip!/╜¡╦╒/┴1⁄4╘╞╕█╩╨/└┤╫╘╣1⁄2╓┌║┼í╛┬φ┐╦╩2╛▌═°í┐_2017.docx]

各位代表：

现在，我代表连云港市人民政府向大会报告工作，请予审议，并请各位政协委员提出意见。

过去五年工作回顾

过去的五年，是我市抢抓国家一带一路建设和江苏沿海开发战略机遇，推动经济社会发展取得重大成就的五年。在市委的坚强领导下，我们全面贯彻党的十八大和十八届三中四中五中六中全会精神，深入落实习近平总书记系列重要讲话精神，自觉践行五大发展理念，坚持稳中求进工作总基调，主动适应经济发展新常态，统筹做好改革发展稳定各项工作，较好完成了本届政府工作目标任务。全市GDP达到2376.48亿元，年均增长10.6%；一般公共预算收入达到211.47亿元，固定资产投资达到2385.16亿元，社会消费品零售总额达到933.31亿元，城乡居民收入年均分别增长8.3%、9%。上一轮脱贫奔小康工程圆满收官，全面小康社会建设迈出坚实步伐。

紧抓第一要务，经济发展稳中向好。深入推进工业立市、产业强市，坚持一二三产业并举，大中小项目齐上，国资外资民资同抓，市县区乡镇联动，不断扩大项目投入。装备制造、石化产业产值突破千亿，百亿特色产业达到12个。连云港跻身国家规划建设的七大石化产业基地。三新一高为代表的战略性新兴产业持续快速发展，高新技术产业占工业总产值比重达到39.1%。实施企业培育三大行动计划，规模以上工业企业净增466家，新增上市企业3家、新三板挂牌企业14家。服务业加快发展，商贸流通业提档升级，苏宁广场、万达广场、东海水晶城等商业综合体人气集聚，民主路、盐河巷、连云老街等传统街区活力激发。电子商务快速发展，被认定为省跨境试点城市，海州智慧物流园成为省级重点物流基地。金融服务实体经济力度加大，社会融资规模达到1970亿元，引进招商银行、国泰君安等金融机构24家。花果山创成国家5A级景区，新增国家4A级景区7家，旅游业年均收入突破300亿元。农业现代化迈上新台阶，粮食生产连年丰收，高效农业、渔业面积分别达到116万亩和52万亩，建成高标准农田315万亩。外向型农业全省领先，一带一路农业国际合作示范区建设稳步推进，赣榆获评国家级现代农业示范区。国家地理标志保护产品达10个。省内首个农产品公用品牌连天下启用。

完善交通设施，综合枢纽功能显著提升。30万吨级航道一期工程建成，二期工程获批建设，30万吨级矿石码头投用。赣榆、徐圩、灌河港区开港运营。港口总吞吐量达到2.2亿吨、集装箱运量465万标箱。新开通19条远近洋航线。中亚、中欧国际班列运行效率国内领先。疏港航道、盐河航道建成通航，内河航道实现千吨级船舶直通京杭大运河，多式联运项目入选国家示范工程。连徐高铁开工建设，连盐、连青、连淮扬镇铁路加快推进，铁路综合客运枢纽正式开工。新改建国省干线公路345公里、农村公路2994公里，形成两纵一横高速公路网和四纵五横高等级干线公路网。白塔埠机场年旅客吞吐量达到85万人次，新机场建设前期工作取得实质性进展。重大交通基础设施的日益完善，为连云港加快发展提供了更有力的支撑。

深化改革开放，发展动能得到有效激发。深入推进供给侧结构性改革，小钢铁、小化工去产能成效明显，商品住房去化周期大幅缩短，营改增全面实施，累计为工业企业降低成本约90亿元。政府性债务得到有效管控，金融风险防范处置机制更加健全。全面取消非行政许可审批事项，实施企业和个体工商户新登记制度。不动产登记制度全面推行。顺利改革重组六大国企集团。基本药物制度全面建立，公立医院综合改革稳步推进。深入实施创新驱动战略，高新区升级为国家高新技术产业开发区，获批建设国家知识产权试点城市和国家农业科技园区，全社会研发投入占GDP比重提高到1.78%，科技进步贡献率提高到53%，高新技术企业增至226家。海陆双向开放格局加快构建，被确定为新亚欧大陆桥经济走廊重要节点城市、中哈物流中转基地、上合组织出海基地。中哈物流合作基地一园三区布局初步形成，上合组织国际物流园建设拉开框架。三互三个一口岸改革深入推进，启运港退税政策获批实施，成为中韩陆海联运扩大试点口岸。成功举办两届新亚欧大陆桥安全走廊国际执法合作论坛，永久会址和培训研究中心落户我市。承办三届连博会、第十八届江苏农洽会。外向型经济逐步增强，累计实际利用外资39亿美元，完成外贸进出口2357亿元，新批境外投资企业82家。全省首家保税商品展示交易中心落户。

注重统筹发展，城乡面貌焕然一新。干净、方便、顺畅连云港建设初见成效，棚户区、老旧小区、城镇危旧房改造力度加大，整治低洼易涝片区24个、背街小巷2300条，市区生活垃圾无害化处理率达100%。城市饮用水双源双线工程建成投用，百万市民喝上放心水。新建改造供热、供气、给排水管网300公里，建成220千伏独立分区电网。海滨大道、徐新公路等道路通车。城市快速公交、公共自行车等绿色出行网络加快完善。市工业展览中心、民政公共服务中心、社会福利中心等公共服务设施投用。13个乡镇列入省级以上重点镇培育计划，创建三星级康居示范村47个、美丽乡村29个。深入推进环境综合整治，秸秆综禁成效明显，化工园区整治力度加大，空气优良率全省领先，建成区绿地率达37.6%。在全国地级市中率先完成战略环评试点。东海、赣榆通过国家级生态县区验收，市开发区获批国家生态工业示范区。创成全国绿化模范城市、国家园林城市和国家节水型城市。去年，我市集中力量补短板，众志成城齐攻坚，国家卫生城市创建圆满通过技术评估。

致力改善民生，群众获得感明显增强。实施教育强基工程，在苏北率先实现教育基本现代化，新建海州高级中学等中小学48所，改造中小学校舍275万平方米，淮海工学院硕士点获批，康达学院建成办学，江苏财会职业学院升格。建成市一院新院区等一批重点项目，市属医院全部达三级标准，分级诊疗加快推进，全市所有乡镇和街道实现了基层卫生机构全覆盖。实施文化惠民工程，率先完成省公共文化服务标准化试点城市建设目标，万人拥有公共文化服务面积1300平方米，年均惠民演出、展览、放映达2万余场。全面建成省公共体育服务示范区，形成城市10分钟体育健身圈，人均体育场地设施面积苏北领先。实施就业创业促进工程，累计新增城镇就业人员38.7万人、农村劳动力转移就业17.7万人，高校毕业生就业率稳定在90%以上。城镇职工五险参保率稳定在95%以上，大病保险实现应保尽保，城乡低保、养老金、残疾人补助等保障标准明显提高，每千名老人拥有床位35.6张。基本建成各类保障房2.83万套，新增住房公积金受益人群3万人，更多困难群众住上了安全合适的住房。创新和加强社会治理，一委三会社会治理模式在全省推广，建成省级以上民主法治示范村（社区）635个。公民科学素质明显提升，江苏海洋发展研究院等智库平台建设取得实效。雷锋车组、最美法官姜霜菊、开山岛夫妻哨、谢芳丽家庭等成为全国先进典型。龚翔宇和队友一起获得奥运会冠军，董贵军成功登顶珠峰，新海实验中学代表队获得中国汉字听写大会全国总冠军，极大地振奋了港城人民的精神。全国双拥模范城实现七连冠。社会治安、安全生产、食品药品安全和信访工作持续稳定。自觉接受人大的工作监督、法律监督和政协的民主监督，办理人大代表建议722件、政协委员提案1643件，按期办复率100%、满意率达99.7%。民族、宗教、档案、史志、民防、地震、气象、妇女、儿童、青少年、老龄、残疾人、红十字、慈善事业等取得新进步。

发展成就来之不易，实践经验弥足珍贵。回顾五年来的奋斗历程，我们深刻体会到：一要竭力抓发展。坚定不移实施产业强市战略，心无旁骛抓项目，全力以赴抓招商，一着不让抓创新，千方百计提振港城经济，夯实加快发展、后发先至的物质基础。二要全力惠民生。坚持将改善民生作为一切工作的出发点和落脚点，持续发力、久久为功推进民生工程，每年办成一批惠民实事，为人民生活小康、身心健康、幸福安康全力而为。三要量力搞建设。坚持规划引领，扎实做好打基础、利长远的工作，加快完善城乡基础设施，不断提升城镇综合承载力和集聚辐射力，共同打造美好家园。四要奋力抓落实。坚持埋头苦干、真抓实干、敢抓敢干、善谋善干，始终保持昂扬向上的精神状态，以抓铁有痕、踏石留印的劲头抓落实，真正做到言必信、行必果。五要努力抓提升。抬高标杆、提升标准，加快职能转变，狠抓效能提升，持续推进政府自身建设，不断优化发展环境，更好地服务群众、服务企业、服务基层、推动发展。

各位代表，过去五年的发展成绩，是省委、省政府和市委坚强领导，市人大、市政协和社会各界监督支持，各级各部门和全市人民团结奋斗的结果。在此，我代表市人民政府，向全市人民，向各位人大代表、政协委员，向各民主党派、工商联、人民团体和社会各界人士，向驻连部队、武警官兵、公安干警，向为连云港改革发展稳定作出贡献的驻连部省属单位，向所有关心支持连云港发展的海内外朋友，表示衷心的感谢和崇高的敬意！

在充分肯定成绩的同时，我们也清醒地看到，我市经济社会发展中还存在一些不足：经济发展仍不充分，产业短板依然突出；港口核心战略资源作用发挥不够，港产城融合发展需要加强；创业创新氛围不浓，人才短缺仍然是制约发展的瓶颈；民生社会事业发展与群众期望还有一定差距；政府效能有待提升，发展环境需要进一步优化。对此，我们将坚持问题导向，采取更加得力措施，切实加以解决。

今后五年奋斗目标和重点任务

今后五年，是我市高水平全面建成小康社会的决胜阶段，是加快建成我国沿海新型临港产业基地、一带一路重要节点城市的关键时期。根据市第十二次党代会部署，今后五年政府工作的指导思想是：高举中国特色社会主义伟大旗帜，深入贯彻落实党的十八大以来中央各项决策部署、习近平总书记系列重要讲话特别是视察江苏重要讲话精神，紧紧围绕五位一体总体布局和四个全面战略布局，践行五大发展理念，抓住用好多重叠加战略机遇，深化供给侧结构性改革，聚力创新，聚焦富民，大力推进沿海发展五大工程，全面加快产业强市步伐，高水平全面建成小康社会，努力建设经济强、百姓富、环境美、社会文明程度高的连云港，奋力开创国际化海港中心城市建设新局面。

今后五年我市经济社会发展的主要目标是：地区生产总值、城乡居民收入保持两位数增长，工业总产值超1万亿元，人均GDP超1万美元，一般公共预算收入年均增长7.5%左右。全市综合经济实力迈上新台阶，各项社会事业更加繁荣，生态环境更加优美，人民生活更加幸福。

--加快崛起沿海新型临港产业基地。坚定不移实施以港兴市、产业强市战略，做大做强现有企业，务实高效组织招商，完成有效产业投入4000亿元以上，构筑以临港大工业和战略性新兴产业为主导、一二三产业融合发展的现代产业体系，重点培育产出超千亿企业1家、过500亿企业2家、200亿企业10家、100亿企业10家、拟上市及挂牌企业80家、科技小巨人企业90家，基本建成国内一流的新材料产业基地、全球知名的新医药产业基地和世界级石化产业基地。

--建设全国重要综合交通枢纽。深入实施水运主通道建设，建成30万吨级航道、30万吨级原油码头，新增万吨级及以上泊位30个，实现一体两翼港区深水航道通航，打造区域性国际航运中心。连徐、连盐、连青、连淮扬镇4条高铁建成通车。建成区域性国际空港，花果山国际机场建成使用，实现一类口岸开放。形成连接京杭大运河、长江的内河干线航道网。建成面向周边地区的七纵十横干线公路。构筑现代化的海陆空铁水立体交通体系，凸显沟通南北、连接东西的国际中转战略节点优势。

--构筑一带一路双向开放门户。放大向东开放优势，做好向西开放文章，一带一路交汇点核心区先导区建设取得明显成效，基本建成中哈物流合作基地和上合组织出海基地，高效运营亚欧国际出口班列。全面推进国家级经济技术开发区转型升级，加快建设国家东中西区域合作示范区，完善区域性国际商务中心综合服务功能，扎实推进自贸区申建工作，健全区域合作和对外开放体制机制，强化对外经贸人文交流合作，打造开放发展新高地，全面提升连云港国际化水平。

--打造区域性科技创新中心。国家创新型城市建设取得阶段性成果，全社会研发投入占GDP比重达到2.5%，科技进步贡献率达到60%。支持优势企业提升自主创新能力，加速优势产业重大技术攻关与产业化，力争在若干领域达到国内乃至世界先进水平。培育国家级高新技术产业基地5个，国内一流的重大科技载体平台20家，建成国家知识产权示范城市和国家农业科技园区，高新区进入国家创新型特色园区行列。大力推动大众创业、万众创新，让创新创业无处不在、蔚然成风。

--增创生态文明优势竞争力。充分发挥自然生态本底优势，认真落实全国首家战略环评试点地级市成果，深入推进绿色发展、循环发展、低碳发展。空气环境质量优良天数比例达到85%，彻底消除城市河道黑臭水体，地表水环境质量达到或优于三类比例超过70%，集中式饮用水水源水质优良比例达到100%，耕地土壤环境质量总体保持稳定，生物多样性得到有效保护，林木覆盖率达到30%，城镇绿化覆盖率达到40%，打造生态海滨城市，构建蔚蓝活力海州湾。

--实现民生社会事业全面进步。把就业创业作为民生之本，加快发展经济，创造更多机会，确保每年新增城镇就业6万人以上，让更多乡亲返乡创业，让连云港逐步实现本地充分就业，农村低收入人口全部脱贫。加快教育、医疗、社保、文化、公共安全等普惠性社会事业发展，提供更加均衡、更高质量的公共产品和公共服务，更好地满足人民群众的需求。更大力度推进干净、方便、顺畅、健康、安全连云港建设，扎实办好民生实事，让人民群众得到更多的实惠和便捷，感受到生活每年都有新变化。争创全国文明城市，推动社会文明程度整体提升。促进军民融合深度发展，深化国防动员和双拥共建，巩固发展军政军民团结的良好局面。努力建成体现高水平要求、惠及全市人民的小康社会。

2017年主要任务

2017年是喜迎十九大胜利召开之年，是全面落实市第十二次党代会部署的开局之年，也是加快建设我国沿海新型临港产业基地的突破之年。全市经济社会发展的主要预期目标是：地区生产总值增长8%，一般公共预算收入同口径增长5%，规模以上固定资产投资增长10%以上，社会消费品零售总额增长10.5%，实际利用外资增长18%。外贸进出口实现正增长，城乡居民收入与地区生产总值保持同步增长，全社会研发投入占GDP比重达到1.8%，居民消费价格指数不高于省控线，节能减排确保完成省定任务。

一、加快发展实体经济，推进产业转型升级

做大做强工业经济。深入推进企业培育三大行动计划，加大技术改造、兼并重组和清费减负治乱力度，完善中小企业公共服务体系，实施精准政策扶持，力争产值过百亿企业达到10家，进入上市辅导期企业2家，新培育科技小巨人企业20家。实施投资亿元以上产业项目260个，力争当年投入700亿元以上。全力突破新型临港产业，产业规模达到2600亿元。推进珠江钢管项目加快建设，推动盛虹炼化项目尽快开工，促进一批临港重大工业项目落地。培育壮大新兴产业，建设省原创化学药创新中心，争创省中医药制造业创新中心、国家医药产业新型工业化示范基地，带动医药产业集群发展。发挥碳纤维、光伏材料、风电领域优势企业带动作用，推进国家级碳纤维复合材料试验公共服务平台建设，加快杜钟新奥神差别化氨纶纤维、弘扬石英外包管及加热管等项目建设，进一步壮大新材料产业规模。加快建设华能灌云300兆瓦风电场、正崴新能源等项目，田湾核电3号机组工程完工，开工建设抽水蓄能电站。加快发展智能装备、工程装备、海洋生物、海工装备等新兴产业。大力推动传统产业转型发展，实施装备升级和互联网提升计划，建设大数据中心，新培育省级以上互联网与工业融合创新示范试点企业和两化融合贯标试点企业10家。深入推进质量强市，争创省级名牌产品10个。加速建筑产业现代化，力争建筑业特级资质实现突破，建筑业总产值达到850亿元。

大力发展现代服务业。拓展生产性服务业，实施康缘仓储物流、天马快递物流园、苏宁云仓等项目，培育壮大连云港电子口岸、跨境电商、新丝路物联网等一批互联网平台，设立大宗商品交易中心、东海水晶交易中心，丰富商贸流通新业态。新增社会融资规模580亿元，新引进华夏银行等金融机构5家，新增新三板及区域性股权交易中心挂牌企业10家，搭建创投产业基金平台2个。全面提升生活性服务业，实施民主路二期、金鹰商业综合体、利群综合体等15个重点商贸设施项目，改造白虎山小商品市场，建成瀛洲路农贸市场。加快建设家庭服务网络平台，重点培育一批管理规范、运作良好的家庭服务企业，创新家政服务产品，提升家庭服务品质。丰富旅游发展业态，推进花果山山南片区开发、云台山民俗、海上旅游公交等重点项目建设，积极发展海洋休闲、海岛旅游、海洋体育等走向大海、亲近大海的滨海旅游新业态，推动传统观光旅游向现代休闲体验度假旅游转变。推动杰瑞科技创意产业园提档升级，支持鸿奥科技、峰之谷等企业开发西游记动漫网游产品，加快成长性文化创意产业发展。依托良好的海滨休闲环境和医药产业基础，重点推进省海滨康复医院、健康产业园、中医老年养护院等项目建设，推动医养融合的健康产业、养老产业加快发展。

加快建设现代农业。落实粮食安全责任制，推进生产全程机械化，粮食总产保持稳定。加快农业产业结构调整，发展优质稻米产业，完成设施农业3万亩、现代渔业1万亩、优质林果茶1万亩。扎实开展农产品质量安全市创建，创成省级农产品质量安全县1个，提升连天下农产品区域公用品牌竞争力，新增市级农业标准化示范区3个，创建生态循环农业示范区10个，制定市级农业地方标准15个，新增三品农产品100个。农产品出口额达到5亿美元。实施龙头企业数量倍增计划，新增市级以上龙头企业40家。加快推进达博澳牛隔离场和裕灌二期发酵槽等项目。新培育示范经营大户、家庭农场、农民专业合作社各100家。新增高标准农田15万亩、高效节水灌溉5万亩，培育电子商务产业园10个、农产品特色网店100个。

二、扩大海陆双向开放，构筑合作共赢高地

增强港口综合功能。开工建设30万吨级航道二期和30万吨级原油码头工程，建成徐圩港区防波堤、赣榆港区防波堤二期延伸段主体工程。建设旗台东疏港公路互通立交、赣榆临港产业区铁路专用线等工程，完善烧香河三级航道功能，实施灌河航道整治。新增集装箱航线3条，运营好美西南、波斯湾2条始发港航线，海河联运突破800万吨。加密中亚、中欧国际班列，巩固全国领先地位。港口集团财务公司开业运营。力争两翼港区口岸开放获批。强化资源整合，深化口岸通关三互三个一改革，建设国际贸易单一窗口和多式联运监管中心，打造绿色智慧港口，加快形成比较优势。

加快两基地建设。中哈物流合作基地散粮筒仓项目建成投产，哈国小麦过境中转业务全面运行，物流场站年均货运量突破25万标箱。加强与中远海运、哈国铁、中铁等国际知名企业战略合作，推进新疆霍尔果斯物流场站、哈国霍尔果斯-东门经济特区无水港项目建设。上合组织物流园开工建设中外运物流中心工程，加快推进铁路装卸场站、保税仓库项目，海运快件项目、智慧物流信息中心建成投用。

深入推进开放合作。突出重点区域、重点产业、重点项目，强化产业链招商、专题招商和以商招商，强攻突破龙头型重大产业项目，不断提升引进项目的质量和水平。全年实际利用外资6.5亿美元以上。坚持招商引资与招才引智互动并进，着力引进更多高层次人才。加快企业走出去步伐，建立境外能源资源合作基地。实施外贸进出口基地和名优品牌培育计划，保持外贸平稳增长。高标准建设一带一路农业国际合作示范区，争创国家农业对外开放合作试验区。推进大陆桥国际执法安全合作永久会址建设。积极推动一带一路沿线国家设立商务代表机构，精心筹办商务品牌展会，密切对外经贸合作和人文交流。

三、提升城市功能品质，彰显山海特色风貌

强化基础设施建设。推进连徐、连青、连淮扬镇等铁路以及新机场建设，建成连盐铁路，完成铁路综合客运枢纽一期工程。204国道连云港段全线通车，建成402省道赣榆西段、267省道东海南段。启动城市组团间道路快速化改造，新建改造临洪大道等市政道路50条，打通断头路5条，开工建设宿徐路，实施云台山隧道整体加固工程。完成城市轨道交通线网规划。加强城市地下空间综合利用开发，加快徐圩、赣榆综合管廊建设，依法合理利用人防工程。全面推进海绵城市试点项目。启动第三水厂扩建，完成海州水厂深度处理，实施徐圩应急备用水源工程。新改建供水管网20公里、燃气管道20公里。加快电网特高压工程建设，建成110千伏及以上输变电工程15个。

加快城建提档升级。巩固扩大创卫成果，建立健全城市治理长效机制。全面实施三改一拆两清理行动，市区完成145万平方米棚户区、155万平方米城中村和60个老旧小区改造，整治老城区背街小巷150条，拆除违建18万平方米。大力实施河道、公路、铁路沿线环境综合治理。推行江苏交通一卡通，新辟优化公交线路10条，公交出行分担率提高到24%，市区新施划停车泊位2000个，拓展公共自行车、绿道慢行系统功能。开展公共标识导向系统建设，优化调整高速公路出入口交通标志牌。健全落实交通影响评价机制。推进高铁商圈开发，加快丰惠广场、金融新天地等大型商业设施建设，建成科技创业城、新世界文化城。完善城市管理智能化监控平台，实现城区网格化管理全覆盖。推进智慧物业二期工程建设，培育物业标准化服务示范企业20家、示范住宅小区30个。

提升城市特色品位。加强城市设计，强化建筑界面、风格色彩控制引导，打造魅力独特的城市面孔。彰显城市融山透绿自然之美，规划建设园博园、动物园、西凤凰山公园，建成新丝路零点公园。实施清水进城工程，加快龙尾河、玉带河、蔷薇河等生态廊道建设，打造沿河绿色风光带。高水平规划建设临洪河口省级湿地公园。精心打造朐山书院、君堂文博苑等一批地域特色项目，推进海州古城、南城古街等修复，促进历史遗存与现代生活有机融合。

四、统筹全域一体发展，促进城乡融合互动

提升县域发展水平。突出工业主体地位，激发县域版块活力。东海重点发展硅资源加工、新型建材和食品工业，灌云加快发展临港工业、港口物流业，灌南优化发展精细化工以及特种钢产业，赣榆聚焦发展石化、钢铁、装备制造和海洋产业。坚持宜工则工、宜农则农、宜商则商、宜游则游，重点培育一批特色产业集群，推动农村一二三产业融合发展，加快壮大镇村经济。深化农村土地三权分置改革，开展多种形式的股份合作，稳妥推进集体经营性建设用地入市、农民住房财产权抵押担保转让等试点，实现农村集体产权流转交易服务平台县域全覆盖。探索建立农业农村发展用地保障机制，盘活利用存量集体建设用地、空闲农房及宅基地。

推进新型城镇化。加快县城建设，强化功能配套，提高承载能力，打造生态宜居城市。以产业特色鲜明、人文气息浓厚、生态环境优美为导向，着力规划建设一批特色小镇。实施镇区门户、道路、绿化改造提升工程，建成农村公路200公里，改造交通桥梁20座，新建改造水利农桥478座。加强城乡污水管网和小型生态处理设施建设，完善城乡生活垃圾收转运体系，乡镇所在地垃圾无害化处理率达100%、农村达80%以上。治理改造城镇危旧房8.5万平方米。改造农村危旧房1200户。大力开展镇村环境整治提升行动，建设美丽乡村示范片区6个。深入实施病险水利工程除险加固、农村河道疏浚、小农水重点县等工程，巩固提升农村饮水安全，新增农村饮水受益人口8.6万人。

实施精准扶贫精准脱贫。以石梁河、沂河淌两片区为重点，加快实施羽青公路等235个重点扶贫项目。投入2.45亿元实施水库移民后扶项目。加快推进76个产业发展重点项目，建设市级扶贫产业园5个。全力推进教育、健康等专项扶贫行动，帮助低收入农户解决子女上学、看病等困难。在全省率先试行扶贫大病特惠保保险工作。规范农民资金互助合作社运作，积极引导信贷等金融要素投入贫困地区，发放各类扶贫贷款5亿元。年内实现10万低收入人口脱贫、50个经济薄弱村达新八有标准，农村低保月保障标准不低于410元。

五、深入推进改革创新，不断增强发展活力

加大供给侧结构性改革力度。落实好去产能、去库存、去杠杆、降成本、补短板各项工作，重组化工企业15家，化解船舶产能20万载重吨。强化土地供应计划供给，控制商品房、商品住宅去化周期。积极稳妥降低企业杠杆率，非金融企业直接融资占社会融资规模比重达到37%，为实体经济企业减少成本20亿元。优化金融生态环境，有效防范金融风险，确保不良贷款余额和不良贷款率实现双降。加大补短板力度，实施基础设施建设、民生保障、生态建设等领域重点补短板项目130个。

抓好重点领域改革。把行政审批制度改革作为深化放管服改革的重要抓手，全面推进两证整合一照一码登记制度改革，推行容缺预审多评合一集中审批联合验收等措施，建立完善以权力清单为核心的阳光高效审批体制，压缩服务流程，提速项目审批，方便群众办事。制定实施中介服务管理办法，公布中介服务事项清单，136家行业协会商会与行政机关脱钩。抓好改革试点工作任务落实，加快推进11项国家级、13项省级试点改革和11项自主创新改革事项，制定切实可行的工作方案，明确路线图和时间表，积极探索实践，及时总结推广经验。统筹推进其他各项改革，完成10家以上市属国企二、三级企业混合所有制改革，完善51个重要子公司法人治理结构。深度整合财政专项资金，管好用好各类基金。完成企事业单位公务用车制度改革。

聚力科技创新。推动国家高新区争先进位，规划建设智能制造加速器、中船重工创新产业园，创建省级科技服务业特色基地。新建众创空间、星创天地5家以上。支持中科院能动中心建设国家重大科技项目。着力培育企业创新主体，高新技术企业总数突破260家，新增省级企业研发机构20个，培育市级科技服务站30个。实施重大科技创新与成果转化项目50项，开发高新技术产品200项，高新技术产值突破2500亿元。科技进步贡献率达到54%。国家知识产权试点城市通过验收，万人发明专利拥有量达到5件。发放苏科贷2亿元以上。新增国家千人计划人才5名，新引进各类高端人才500名。

六、加强生态文明建设，优化人居发展环境

推进绿色发展。控制能源消耗、水资源消耗、建设用地总量和强度，促进资源节约集约利用。完成30项重点节能项目，50家企业完成清洁生产审核。完成省级以上开发园区循环化改造任务，创建生态工业园区6个、循环农业示范区10个。推广绿色建筑和建材，新增二星级绿色建筑40万平方米。倡导绿色服务和消费，推广应用新能源汽车，建设电动汽车快充站100个，清洁能源和新能源公交车比例达到80%。稳步推进排污权交易制度、一企一档环境管理制度改革。健全生态制度体系，完善绿色发展评估制度。

强化环境治理。深入开展两减六治三提升专项行动，解决一批影响环境质量的突出问题。推行无煤区建设，淘汰城区10蒸吨及以下燃煤锅炉，完成建成区内燃煤小锅炉整治。全面落实河长制，促进五水共治常态化，市区整治黑臭水体5条。完成钢铁企业脱硫，推动新海发电、虹洋热电等燃煤发电机组超低排放改造，搬迁德邦、泰乐、双菱等化工企业。强化城市扬尘常态化管控。推进黄标车等交通污染防治。搬迁关闭禁养区内畜禽养殖场。狠抓化工园区专项整治，坚决淘汰落后化工产能，关停一批、转移一批、升级一批和重组一批化工企业，实现园区河道水质达标、空气无异味，完成卫生防护距离内居民搬迁。强化环保基础设施，建设赣榆新城污水处理厂，建成徐圩东港污水处理厂，提标改造城市污水处理厂5家。华能、亚邦等热电联产项目建成并实现供热管网全覆盖。建成光大环保、丰益精细化学等危废焚烧项目3个，年处置能力新增4.5万吨。

加强生态保护修复。灌云、灌南创成省级生态县。生态红线保护区占国土面积比例提高到23%。整治修复墟沟林场、黄草沟和朝阳西山等采石塘口。完成造林8万亩，山体绿化及林相改造2万亩，培育珍贵树木600万株。推进海州湾海洋公园、海洋牧场示范区、海洋生态补偿工程。启动埒子口治理、新一轮海堤增补完善等工程，保障近岸海域生态安全。

七、持续保障改善民生，致力增进人民福祉

聚焦就业创业富民。按照政府搭建平台、平台聚集资源、资源服务创业的思路，整合建设一批创业创新园区，引导在外务工人员返乡创业。实施职业技能提升计划，开发紧缺职业培训包，开展城乡劳动者技能培训22万人次，新增城镇就业6万人以上，确保城乡零就业、零转移家庭动态为零，城镇登记失业率控制在3%以内。加大就业困难人员援助，帮扶就业2000人。新增私营企业1万户、个体工商户2万户。

加快社会事业发展。全市国家教育信息化达标率达82%，赣榆通过教育现代化省级示范县区评估。积极发展普惠优质幼儿园，规范民办幼儿园办园秩序。新建中小学、幼儿园33所，改造校舍40万平方米。实施乡村师资素质提升工程，培训教师5万人次。支持连云港师专升格本科院校、淮海工学院创建江苏海洋大学。新建市妇幼保健中心，改扩建市中医院，新增省级示范乡镇卫生院、村卫生室20个。推行医联体和分级诊疗制度，家庭医生签约服务覆盖率达30%以上。推进健康连云港建设。完善公共体育文化服务体系，加快建设市全民健身活动中心，建设升级城乡体育场所72个。完成市文化馆、美术馆改造，建成花果山国家地质公园，启动市地情馆建设，搬迁市档案馆，完成市县区二轮修志。组织开展广场文化活动240场次，举办各类群众性演出、展览展映2万场次，组织办好连云港之夏、西游记文化节等活动。启动市殡仪馆搬迁工作。

提高社会保障水平。扩大参保覆盖范围，城镇职工五险平均新增参保1万人以上，城乡居保参保率达98%以上。城乡居民医保财政补助标准提高到人均470元以上，实现医疗保险异地联网结算。建设保障性住房58万平方米，建成公共租赁住房1130套。统筹实施残疾人补贴、医疗救助、慈善救助、特困职工救助等精准救助，有效保障困难群众基本生活。建立困境儿童社会救助保护制度，加强流浪乞讨人员救助管理，推进康复医院建设。提升养老服务水平，新建医养融合型养老机构2个，建成标准化居家养老服务中心（站）30个，新增护理型养老床位600张，市级智慧养老服务平台投入使用。

加强和创新社会治理。深入开展精神文明建设活动，提升公民文明素质，实施全民科学素质行动计划。全面推广一委三会社区治理模式，深入推进三社联动，政社互动实现全覆盖。完成村（居）委会换届。推广文昌社区、院前社区互联网便民服务新模式。加强法治连云港建设，落实七五普法规划，省级民主法治示范村（社区）创建率达40%。深化最安全城市建设，健全完善社会治安防控体系，提高突发事件应急处置能力。依法规范信访秩序，推进信访法治化。加强食品药品安全监管，开展公路治超专项行动，强化安全生产责任和管理制度落实，防范遏制重特大安全事故。加强政府公共服务平台建设，规范健全政府服务体系。提升12345政府热线服务水平，打造连云港发布智慧连云港政务新媒体。推动军民融合发展，积极支持军队调整改革，扎实做好征兵和双拥共建工作。推动民族、宗教、地震、气象、关心下一代、老龄、红十字、慈善等事业再上新台阶。

加强政府自身建设

新形势新任务，新起点新征程，对加强政府自身建设提出了更高要求。我们必须切实增强宗旨意识、使命意识、担当意识、法治意识，全面推进服务型、效率型、务实型、法治型、廉洁型政府建设，为改革发展稳定各项事业提供坚实保障。

（一）转作风优服务。大力发扬真抓实干的优良作风，做到思想务实、工作扎实、举措求实，不搞形式主义，不做表面文章，不搞虚假增长，绝不搞劳民伤财的形象工程。强化主动服务，深入企业，深入基层，深入群众，接地气、知民情、察民盼，扎扎实实解决实际问题。

（二）提效能促发展。加快政府职能转变，理顺政府运行体制机制，提高办事效率，降低行政成本，为企业发展松绑、为群众办事解绊、为基层开展工作扩权，构建亲清政商关系，打造便捷高效的政务环境。

（三）强责任敢担当。大力倡导担当精神，遇事不避难，有难勇往前，做埋头苦干的耕耘者、真抓实干的闯滩者、敢想敢干的冲锋者、善谋善干的改革者。建立健全责任机制、激励机制、容错保护机制，让想干事、干成事的有空间，让怕担当、不作为的没市场，为担当者担当、负责者负责。

（四）讲法治重诚信。推进民主决策，加强依法行政，严格规范执法，做到法定职责必须为，法无授权不可为，自觉接受人大法律监督、政协民主监督和社会舆论监督，确保权力在法治轨道上运行。推进政务公开，强化有效监督，坚持践诺守信，严格失信惩戒，突出重点领域，精准发力整治，切实做到取信于民、取信于企，以政府诚信施政引领全社会诚信建设。

（五）守规矩葆本色。严守政治纪律和政治规矩，落实中央和省、市委部署要求，坚持不懈纠正四风，坚决执行厉行节约规定，严控三公经费。严格落实一岗双责，深入推进党风廉政建设，加强审计监督，强化财政监管，严厉查处违纪违法案件，做到心有所畏、言有所戒、行有所止，树立为民务实清廉形象。

各位代表，高水平全面建成小康社会的接力棒传递在手，实现快速崛起、后发先至的光荣使命重任在肩，人民寄予厚望，组织赋予重托，实干铸就辉煌。让我们紧密团结在以习近平同志为核心的党中央周围，在市委的坚强领导下，同心同德，真抓实干，迎难而上，勇争一流，共创更加幸福美好生活，为谱写好中国梦的连云港篇章而努力奋斗！
